# Abnormal blood pressure response to exercise occurs more frequently in hypertrophic cardiomyopathy patients with the R92W troponin T mutation than in those with myosin mutations

**DOI:** 10.1016/j.hrthm.2009.07.020

**Published:** 2009-11

**Authors:** Marshall Heradien, Miriam Revera, Lize van der Merwe, Althea Goosen, Valerie A. Corfield, Paul A. Brink, Bongani M. Mayosi, Johanna C. Moolman-Smook

**Affiliations:** ⁎ Department of Internal Medicine, University of Stellenbosch Health Sciences Faculty, Tygerberg, South Africa; † Department of Cardiology, Istituto Di Ricovero E Cura A Carattere Scientifico San Matteo Hospital, Pavia, Italy; ‡ Biostatistics Unit, Medical Research Council of South Africa, Tygerberg, South Africa; § Medical Research Council/University of Stellenbosch Centre for Molecular and Cellular Biology, University of Stellenbosch Health Sciences Faculty, Tygerberg, South Africa; ‖ Cardiovascular Genetics Laboratory, Hatter Institute for Cardiovascular Research, Department of Medicine, University of Cape Town, Cape Town, South Africa

**Keywords:** Hypertrophic cardiomyopathy, Abnormal blood pressure response, Survival, Troponin T, Beta-myosin, Genetic mutation, Ca^2+^, calcium, CR, chronotropic response, DBP, diastolic blood pressure, ECG, electrocardiogram, HCM, hypertrophic cardiomyopathy, HR, heart rate, LV, left ventricle, LVM, left ventricular mass, maxLVWT, maximum left ventricular wall thickness, METs, metabolic equivalents, *MYH7*, beta cardiac myosin heavy chain gene, SBP, systolic blood pressure, SCD, sudden cardiac death, *TNNT2*, cardiac troponin T gene

## Abstract

Abnormal blood pressure response to exercise is reported to occur in up to a third of hypertrophic cardiomyopathy (HCM) cases and is associated with an increased risk of death, particularly in the young, but it is not known whether the HCM-causing mutation influences blood pressure response to exercise. The purpose of this article is to ascertain whether the blood pressure response to exercise differs among carriers of the R92W mutation in the cardiac troponin T gene (*TNNT2*), which has been associated with an increased risk of sudden cardiac death in young males; carriers of mutations in the cardiac β-myosin heavy chain gene (*MYH7*); and their noncarrier relatives. Thirty R92W_*TNNT2*_ carriers, 51 *MYH7* mutation carriers, and 68 of their noncarrier relatives were subjected to bicycle ergonometric exercise testing to assess blood pressure response to, as well as heart rate recovery after, exercise. Additional echocardiographic and demographic details were documented for all participants. R92W_*TNNT2*_ carriers demonstrated significantly more abnormal blood pressure responses to exercise (*P* = .021; odds ratio 3.03; confidence interval 1.13–8.12) and smaller increases in systolic blood pressure than *MYH7* mutation carriers or related noncarrier control individuals. Although abnormal blood pressure response occurred at similar frequencies in males in all groups (23%–26%), the percentage of R92W_*TNNT2*_ females with abnormal blood pressure response was 64%, compared with 25% for *MYH7* and 22% for noncarriers. Therefore, these results show that blood pressure response to exercise is influenced by genotype and gender in patients with HCM.

## Introduction

Hypertrophic cardiomyopathy (HCM), a primary cardiac muscle disease with 0.2% prevalence,^1^ is characterized primarily by thickening of the ventricular wall in the absence of other hypertrophy-predisposing conditions^1^ and by an increased risk for sudden cardiac death (SCD), which may relate to changes in cardiac energy and/or Ca^2+^ homeostasis or histopathological changes in the myocardium.^2^ HCM is typically caused by mutations in genes encoding protein components of the cardiac sarcomere (http://genetics.med.harvard.edu/∼seidman/cg3/index.html), with the three most common causes being mutations in the genes encoding beta cardiac myosin heavy chain (*MYH7*), cardiac troponin T (*TNNT2*), and myosin binding protein C. Although the phenotypes resulting from these genetic defects vary greatly, mutations in *TNNT2* in humans^3,4^ and in animal models^5,6^ have frequently resulted in an increased susceptibility to SCD, often in the presence of mild or absent cardiac hypertrophy. We have previously reported an increased frequency of SCD, affecting young males in particular, in HCM families in which the R92W mutation in *TNNT2* segregates,^7^ although the reason for this poor prognosis was not investigated at the time.

Blood pressure response to exercise testing is commonly used as an indicator of risk for SCD in patients with HCM. The failure of blood pressure to increase appropriately in response to exercise has been recorded in 8%–33% of ungenotyped patients with HCM.^8–10^ Abnormal blood pressure responses have been associated with an increase in cardiovascular mortality^8,11^ and have a reported 15% positive predictive value for SCD in HCM.^11^ It is not known whether the blood pressure response to exercise in HCM is influenced by genotype.

We hypothesized that the R92W mutation, which confers a high rate of SCD, may be associated with an abnormal blood pressure response to exercise. Here we investigated this hypothesis by comparing the blood pressure response to exercise in carriers of the R92W_*TNNT2*_ mutation with that of carriers of HCM-causing mutations in the cardiac β-*MYH7* mutations (A797T and R403W) and noncarrier relatives.^12^

## Methods

### Subjects

The study was approved by the Institutional Review Board of the University of Stellenbosch Health Sciences Faculty (N04/03/062). Individuals belonging to consecutively identified HCM families in which either of three founder HCM-causing mutations segregate (R92W_*TNNT2*_, A797T_*MYH7*_, R403W_*MYH7*_)^13^ who did not have pacemakers, who were in sinus rhythm at the time of the exercise test, and who gave written informed consent were included in this study.

A medical and family history was taken, and age, sex, height, and weight as well as sudden death events that had occurred in family members were recorded for each participant. Only sudden deaths occurring in genotyped mutation carriers, or obligate heterozygotes, or individuals with a diagnosis of HCM, or with a history of syncope were considered as SCDs. Blood pressure was taken twice in the sitting position, after 5 minutes of bed rest, and the second measurement was used. Individuals were coded as hypertensive if they on more than one occasion had systolic blood pressure (SBP) ≥140 mmHg or diastolic blood pressure (DBP) ≥90 or were on antihypertensive medication.

### Exercise testing

We performed 12-lead electrocardiography (ECG) on a MAC 1200ST machine after 5 minutes of rest in the supine position and from this determined a resting heart rate (HR) and whether normal sinus rhythm was present. All cardioactive medications were discontinued for at least 5 pharmacokinetic half-lives before exercise testing. Exercise testing was performed by practitioners who were blinded to the genotype status of the participants.

Maximum symptom-limited bicycle ergonometric exercise testing was performed according to the following protocol: eight stages over 24 minutes with incremental increase of 25 W every 2 minutes (25–200 W). Concurrent continuous 12-lead ECG monitoring was performed with the Norav Medical System. SBP and DBP were recorded with a mercury sphygmomanometer by a trained nurse (AG). Blood pressure values were recorded at rest, at 1-minute intervals during exercise, and at 1-minute intervals for 5 minutes during the recovery period.

We recorded the maximal workload achieved (in metabolic equivalents [METs]), total exercise time (in minutes and seconds), the maximum SBP and DBP achieved during exercise, the SBP and DBP and HR at peak exercise and at 1-minute intervals during recovery, and the percentage of predicted maximal HR for age and sex achieved by peak exercise. The predicted maximal HR was calculated as 220 minus age for men and 210 minus age for women.^14^ Chronotropic reserve (CR) was calculated as described elsewhere.^15^

Normal blood pressure response was defined as an increase of at least 20 mmHg in SBP during exercise, with a gradual decline during recovery.^11^ Abnormal blood pressure responses were defined as either (1) an initial increase in SBP with a subsequent fall of >20 mmHg compared with the blood pressure value at peak exercise or a continuous decrease in SBP throughout the exercise test of >20 mmHg compared with resting blood pressure (termed hypotensive responses) or (2) an increase of <20 mmHg in SBP from resting state to peak exercise (termed a flat response).

Exercise was terminated for any of the following reasons: reaching target HR, fatigue, severe dyspnea, significant chest pain, near syncope, or development of arrhythmias.

### Echocardiography

Echocardiography was performed on all participating individuals, using a standardized procedure, by a single experienced echocardiographer (MR) who was blinded to mutation status. A GE Healthcare Vivid7 cardiovascular ultrasound system was used to determine maximum wall thickness at 16 left ventricular (LV) wall segments on M-mode and two-dimensional images, as described elsewhere.^13^ Overall maximum LV wall thickness (maxLVWT) was recorded for each individual.

LV mass (LVM) was calculated using the American Society of Echocardiography formula for estimation of LVM from two-dimensional LV linear dimensions as described elsewhere.^13^ Ejection fraction was determined by the biplane method of discs, using the modified Simpson's rule, while fractional shortening was calculated using LV diastolic and systolic diameters from two-dimensional echocardiography images.^16^ Stroke volume was calculated as

$$SV=\left[7/\left(2.4+\text{LVd}\right)\times \text{L}\text{V}{\text{d}}^{3}\right]-\left[7/\left(2.4+\text{LVs}\right)\times {\text{LVs}}^{3}\right],$$

where LVd = LV end-diastolic diameter and LVs = LV end-systolic diameter. Cardiac output was calculated as CO *=* (SV × HR)/100, where SV is stroke volume and HR is resting HR.

LV outflow obstruction was considered to be present when a peak instantaneous subaortic gradient ≥30 mmHg was estimated with continuous wave Doppler echocardiography under resting conditions.^17^ Mitral regurgitation was graded semiquantitatively (1–4+ scale).^18^

### Statistical analyses

Comparisons were made among three groups consisting of noncarriers, carriers of the R92W_*TNNT2*_, and carriers of either of the *MYH7* mutations. Quantitative variables were quantile normalized before modeling.^19^ As the distribution of age, gender, body size, and resting hemodynamic parameters varied between groups, these factors were adjusted for in statistical comparisons of the hemodynamic responses during exercise between groups. General (quantitative) and generalized (dichotomous, categorical) linear mixed-effects models were used, as required, with adjustment for family relatedness as random factor (R package). *P* <.05 was considered statistically significant.

## Results

Thirty R92W_*TNNT2*_ carriers from seven families, 51 *MYH7* mutation carriers (27 A797T_*MYH7*_ carriers from 10 families, 24 R403W_*MYH7*_ carriers from three families) and 68 of their noncarrier relatives participated in exercise testing. The relevant clinical and demographic characteristics of these individuals are given in Table 1. Total exercise time, workload, and percentage of target HR achieved were similar between groups (Table 2). In the R92W_*TNNT2*_ families, SCD had affected predominantly young males (eight males, age at SCD 22.5 ± 10 years [mean ± standard deviation]; one female, 62 years; Table 3). Among *MYH7* mutation carriers, five male (mean age at SCD 35.8 ± 17.3 years) and three female (mean age at SCD 32 ± 20.2 years) A797T_*MYH7*_ carriers experienced SCD before the study. At least five of the *MYH7* and all R92W_*TNNT2*_ SCDs had occurred during periods of physical activity or emotional excitement (Table 3). One SCD in each of the R92W_*TNNT2*_ and *MYH7* groups occurred in females >40 years.

**Figure 1:**
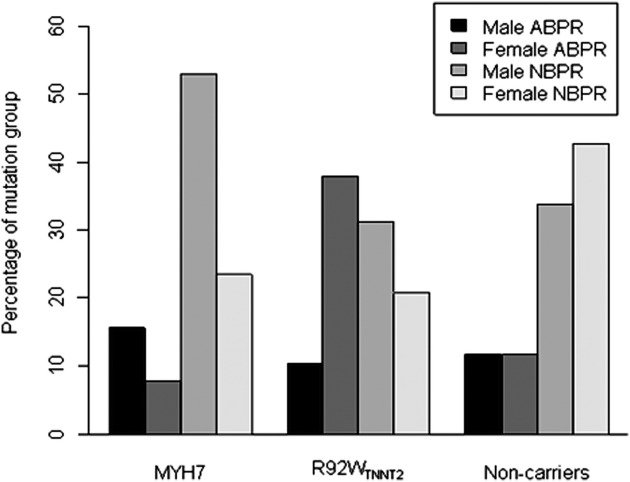
Percentage of males and females within each group demonstrating normal and abnormal blood pressure responses to exercise.

Whereas abnormal blood pressure response to exercise occurred in about a quarter of control individuals or individuals carrying either *MYH7* mutation, this phenomenon was present in significantly more, that is, nearly half, of R92W_*TNNT2*_ carriers (Table 2). No individuals demonstrated a continuous decrease in SBP throughout the exercise test. Flat abnormal blood pressure responses predominated in all groups but were the only type of abnormal blood pressure response in R92W_*TNNT2*_ carriers (Table 2). Statistical analysis indicated that R92W_*TNNT2*_ carriers were 3 times as likely to demonstrate an abnormal blood pressure response to exercise compared with either *MYH7* mutation carriers or noncarrier control individuals (Table 4). This was particularly noticeable for female R92W_*TNNT2*_ carriers (Figure 1); the percentage of females with abnormal blood pressure response for R92W_*TNNT2*_ was 64%, for *MYH7* 25%, and for noncarriers 22%. In contrast, the percentage of males with abnormal blood pressure response was similar for the three groups (R92W_*TNNT2*_ 24%; *MYH7* 23%; noncarriers 26%).

**Figure 2:**
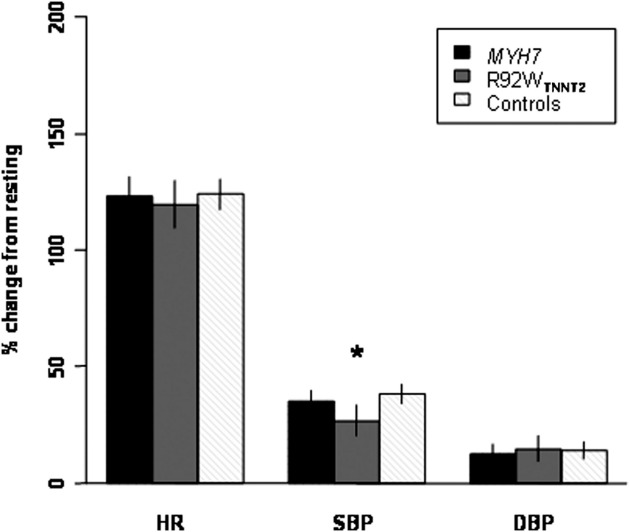
Bar graph of adjusted means and 95% confidence interval of untransformed values, showing percentage change in hemodynamic parameters from resting to peak exercise. *P = .029.

After adjustment for age, sex, resting mean arterial pressure, and resting HR, the effect of exercise on HR and DBP parameters (Table 2) was not different between groups. However, R92W_*TNNT2*_ carriers achieved a significantly lower mean SBP at peak exercise, as well as a smaller mean change (Table 2) and a smaller percentage of change in SBP (Figure 2) between resting and peak exercise, than did noncarriers and *MYH7* mutation carriers.

**Figure 3:**
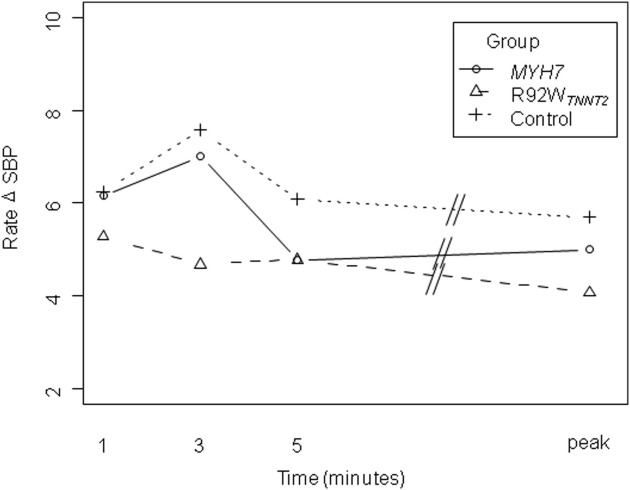
Mean rate of change in SBP in the groups during early exercise and at peak exercise. Values were adjusted for age, sex, body surface area, resting HR, and resting SBP.

Interestingly, we also observed that, while the mean rate of SBP change increased in the first 3 minutes of exercise in the control and *MYH7* groups, R92W_*TNNT2*_ carriers on average demonstrated a slight decrease in the rate at which SBP changed during the first 3 minutes of exercise (Figure 3). However, perhaps owing to the small numbers of individuals involved, this difference was not statistically significant.

## Discussion

Abnormal blood pressure response to exercise has been associated with HCM-related death, with patients <50 years old showing a fourfold increase in premature HCM-related death.^8,11^ However, it is unknown whether abnormal blood pressure response is influenced by the HCM-causing genotype. Here we show that individuals carrying the R92W_*TNNT2*_ mutation are 3 times as likely to experience abnormal blood pressure response upon exercise compared with those carrying either of two *MYH7* mutations or noncarrier controls.

We have previously described an increased frequency of SCD, predominantly affecting young males in their teenage years, in two ancestrally related families in which the R92W_*TNNT2*_ mutation segregates.^7^ In this study, we report SCDs in a further two of five additional R92W_*TNNT2*_ families identified (Table 3); these families have been shown by genetic haplotype analysis (data not shown) to be ancestrally related to the original two R92W_*TNNT2*_ families.^7^ While SCD had occurred in four (57%) of the seven R92W_*TNNT2*_ families, predominantly affecting young men in their teenage years, SCD in *MYH7* families was limited to those in which the A797T_*MYH7*_ mutation segregates (five [50%] of 10 A797T_*MYH7*_ families, 39% of total *MYH7* families) and mostly affected adult individuals (Table 3). Similar to our first report of the clinical picture associated with R92W_*TNNT2*_ carriers, overt hypertrophy was a rare occurrence in the R92W_*TNNT2*_ group^7^ but occurred more frequently in the *MYH7* group (n = 4, all A797T_*MYH7*_ carriers), while syncope occurred at comparable levels in both mutation groups (Table 1).

We found that carriers of the R92W_*TNNT2*_ mutation demonstrate abnormal blood pressure response to exercise significantly more frequently (Tables 2 and 4; *P* = .021, odds ratio = 3.03) and show smaller absolute (ΔSBP, Table 2) and relative (% change from resting SBP, Figure 2) changes in SBP than do carriers of *MYH7* mutations or related noncarrier control individuals. Thus, our study of the largest number of cases with an identical mutation in the *TNNT2* gene to date provides strong evidence for the influence of genotype on hemodynamic response in HCM.

In R92W_*TNNT2*_ carriers, abnormal blood pressure responses were always of the flat type, namely, a failure to increase SBP by >20 mmHg between resting and peak exercise state. This finding, as well as the smaller increases in SBP observed in this study in the R92W_*TNNT2*_ group during exercise (Table 2, Figure 1), supports the results of Sakata et al,^20^ who recently reported that a group consisting of carriers of diverse troponin mutations demonstrated a smaller increase in SBP during exercise than did individuals without troponin mutations.

The mechanisms underlying abnormal blood pressure response to exercise in HCM are not yet completely understood. While some studies indicate that this response is due to an exaggerated decrease in systemic vascular resistance,^9,21^ other studies suggest that impaired cardiac output is responsible for exercise hypotension.^22,23^ Although it remains possible that either or a combination of both mechanisms are at play in subsets of HCM patients,^24^ Sakata et al^20^ found that systemic vascular resistance decreased to a similar extent in the *TNNT2* and non-*TNNT2* groups. They proposed that their findings related to the systolic dysfunction, which would result in decreased cardiac output that developed only in the *TNNT2* group during exercise.

The development of abnormal blood pressure response may also be related to autonomic control, as studies of neurotransmitter levels before and after exercise have also previously indicated that HCM patients manifest a sympathoadrenal imbalance during exercise.^25,26^ The cause of this imbalance is not known, nor is it known whether this imbalance is genotype related, but it has been suggested that the enhanced systolic function that occurs under resting conditions in transgenic mutant *TNNT2* mice,^27^ as well as in prehypertrophic R92W_*TNNT2*_ carriers,^13^ may activate ventricular mechanoreceptors and cause chronic alterations in vagal tone. On the other hand, Kawasaki et al^10^ recently demonstrated that subendocardial ischemia, which occurs in about 50% of HCM patients upon exercise,^23,28^ leads to vagal enhancement in patients with HCM, which may be related to the development of abnormal blood pressure response to exercise.

The preponderance of abnormal blood pressure response in females in the R92W_*TNNT2*_ group might be explained by vagal enhancement, which results from either mechanism proposed above, occurring on the background of the normally predominantly parasympathetically biased cardiac regulation in females.^29^ The preponderance of abnormal blood pressure response in female R92W_*TNNT2*_ carriers, who experience less SCD than their male counterparts, could suggest that this hemodynamic factor is not causally associated with the high frequency of sudden death with exercise that we have observed in the R92W_*TNNT2*_ group. However, in light of previous longitudinal studies indicating an increase in premature HCM-related death among HCM patients demonstrating abnormal blood pressure response to exercise,^8,11^ the survival and abnormal blood pressure response data in this study may suggest that a parasympathetic shift is not well tolerated in the predominantly sympathetically regulated male hearts.^29^

Failure to increase SBP appropriately during exercise is indicative of severe coronary artery disease in the general population^30,31^ and is more marked for those who fail to increase SBP appropriately during the first 3 minutes of exercise rather than later on during exercise.^30^ This is interesting, given our preliminary observation of the rate of change in SBP, which suggests that individuals with the R92W_*TNNT2*_ mutation may fail to increase blood pressure appropriately, particularly early on during exercise (Figure 3).

In contrast to the high rate of abnormal blood pressure response to exercise in the R92W_*TNNT2*_ group, the frequency of abnormal blood pressure response to exercise in the *MYH7* group was similar to that reported for genotype-unknown HCM patients^8,9^ and was equal to that observed in the control group of noncarrier relatives (62% from *MYH7* and 38% from R92W_*TNNT2*_ families). However, the frequency of abnormal blood pressure response to exercise in this control group was much higher than that reported for a non–South African general population (2%–8%).^30^ The reason for this is not immediately clear; however, half of the control individuals with abnormal blood pressure response in this study were overweight or obese (body mass index >25), perhaps indicating a risk for coronary artery disease and silent myocardial ischemia.^32^ Other reported causes of an abnormal blood pressure response to exercise include valvular heart disease, orthostatic hypotension, and the use of drugs such as vasodilators, negative inotropic agents, and diuretics.^30^

## Conclusion

Individuals with R92W_*TNNT2*_ mutations demonstrate abnormal blood pressure response to exercise significantly more frequently than do individuals with *MYH7* mutations or noncarrier controls, indicating that abnormal blood pressure response is influenced by genotype. The preponderance of female R92W_*TNNT2*_ carriers with abnormal blood pressure response, with a lower risk of SCD than affected males, supports the suggestion that abnormal blood pressure response may be associated with a parasympathetic shift in the regulation of cardiac function and that parasympathetic shifts are not well tolerated in predominantly sympathetically regulated male hearts. Although implantable defibrillators are the most effective life-saving therapy available for prevention of SCD in HCM, this option is not widely available outside of developed nations owing to cost. Elucidating the molecular mechanisms underlying SCD in HCM may eventually facilitate development of alternative effective therapeutic options.

## Figures and Tables

**Table 1:** Clinical characteristics of subjects

	*MYH7*	R92W_*TNNT2*_	Controls
N	51	30	68
Males, % (n)	35 (69)	12 (41)	31 (46)
Age, years	38 (17–72)	34 (14–66)	34 (17–63)
Body surface area, kg/m^2^	1.9 (1.3–2.5)	1.7 (1.3–2.1)	1.8 (1.3–2.3)
LVM, g	182 (71–477)	126 (48–290)	122 (58–272)
maxLVWT, mm	13.8 (7.7–38.2)	11.6 (6.7–30.0)	10.1 (7.1–27.0)
Left atrial diameter, mm	35.8 (20.0–65.0)	32.9 (21.0–49.0)	30.6 (22.0–45.0)
LV end-diastolic diameter, mm	44.0 (32.0–52.0)	40.5 (33.0–48.0)	44.0 (37.0–60.0)
LV end-systolic diameter, mm	26.8 (17.0–38.0)	22.7 (16.1–33.2)	28.0 (21.0–44.0)
Ejection fraction, %	70 (57–81)	71 (61–79)	65 (52–75)
SV, mL	83 (33–124)	78 (43–103)	80 (48–133)
Cardiac output at rest	6.2 (2.0–11.3)	5.3 (3.3–8.9)	6.0 (4.2–10.9)
Resting HR, bpm	75 (60–145)	72 (55–118)	78 (48–123)
SBP_resting_, mmHg	120 (80–140)	110 (90–130)	115 (90–160)
DBP_resting_, mmHg	80 (60–100)	70 (60–90)	80 (60–100)
Outflow tract gradient >30 mmHg	0 (0)	0 (0)	0 (0)
Systolic anterior motion of the mitral valve	4 (8)	0 (0)	0 (0)
MR = 1	18 (36)	4 (15)	4 (7)
MR = 2	1 (2)	1 (4)	1 (2)
NYHA 2	8 (16)	3 (11)	2 (3)
Atrial fibrillation	2 (4)	2 (7)	0 (0)
Hypertension diagnosis	7 (14)	0 (0)	2 (3)
maxLVWT ≥30 mm	4 (8)	1 (3)	0 (0)
Syncope	4 (8)	3 (10)	2 (3)
% Families with SCD	39	57	NA

*Note:* Values are given as median (range) for continuous variables and as numbers (percentage) for categorical variables. No individuals were above New York Heart Association (NYHA) class 2 or mitral regurgitation (MR) score 2 (according to Zhogbi et al.^33^ 2003). NA = not applicable.

**Table 2:** Blood pressure and HR response to exercise in R92W_*TNNT2*_ and *MYH7* mutation carriers and noncarrier controls

	*MYH7*	R92W_*TNNT2*_	Controls	*P*
Total exercise time, seconds	528 (96–1104)	379 (266–937)	467 (171–1319)	.339
MET, kcal/min	6.5 (2.3–12.2)	6.5 (3.9–14.7)	6.2 (2.9–12.1)	.660
% HR achieved	96 (74–104)	89 (56–104)	94 (75–108)	.417
%CR	88.0 (32.3–141.2)	83.7 (24.8–143.1)	89.4 (54.1–118.5)	.487
HR_peak_, bpm	171 (129–203)	169 (98–211)	170 (123–197)	.449
ΔHR, bpm	92.0 (10–140)	92.0 (28–136)	90.5 (44–137)	.499
Abnormal blood pressure response, any (%)	12 (24)	14 (48)	16 (24)	.021
Flat (%)	10 (20)	14 (48)	11 (16)	.018
Hypotensive (%)	2 (4)	0 (0)	5 (8)	.357
SBP_peak_, mmHg	160 (120–230)	140 (100–190)	160 (110–220)	.064
ΔSBP, mmHg	35 (0–100)	30 (10–60)	40 (5–100)	.020
DBP_peak_, mmHg	90 (70–120)	90 (50–100)	90 (60–120)	.589
ΔDBP, mmHg	10 (−20–40)	10 (−10–30)	10 (−20–40)	.732

*Note:* Values are given as median (range) for continuous variables and as numbers (percentage) for categorical variables. *P*-values reflect differences between groups for quantile normalized data, adjusted for age, sex, resting mean arterial pressure, and resting HR. Δ = change in parameter between resting and peak exercise; peak = parameter at peak exercise; % HR achieved = % of predicted maximal HR achieved by peak exercise.

**Table 3:** Characteristics of individuals who experienced SCD

Families	Sex	Age, years	Circumstances of SCD
R92W_*TNNT2*_:			
Ped 100	F	62	Physical exertion
Ped 100	M	16	Physical exertion
Ped 100	M	14	Physical exertion
Ped 100	M	36	Physical exertion
Ped 109	M	23	Physical exertion
Ped 137	M	17	Physical exertion
Ped 137	M	15	Physical exertion
Ped 139	M	40	Physical exertion
Ped 139	M	19	Physical exertion
A797T_*MYH7*_:			
Ped 101	F	55	Washing dishes
Ped 101	F	24	Upon return from work
Ped 101	F	17	Emotional exertion
Ped 101	M	22	Physical exertion
Ped 104	M	40	Physical exertion
Ped 131	M	23	Sedentary
Ped 147	M	30	Physical exertion
Ped 158	M	64	Physical exertion

*Note:* F = female; M = male.

**Table 4:** *P*-values and estimated odds ratio (OR) for abnormal blood pressure response in R92W_*TNNT2*_ compared with *MYH7* carriers and noncarrier control individuals, adjusted for age, sex, resting mean arterial pressure, resting HR, and family relatedness

Groups	OR	95% Confidence interval	*P*
*R92W*_*TNNT2*_			
*MYH7*	3.03	1.13, 8.12	.029
*R92W*_*TNNT2*_			
Control	3.03	1.20, 7.68	.021
*MYH7*			
Control	1.00	0.42, 2.37	1.000
